# Epidemiological characteristics and adherence of a cohort of elderly people with HIV/AIDS in the Public Health System

**DOI:** 10.31744/einstein_journal/2022AO6474

**Published:** 2022-03-17

**Authors:** Cristiane Marcos Soares Dias Ferreira, Isabel Cristina Gonçalves Leite

**Affiliations:** 1 Universidade Federal de Juiz de Fora Juiz de Fora MG Brazil Universidade Federal de Juiz de Fora, Juiz de Fora, MG, Brazil.

**Keywords:** Treatment adherence and compliance, HIV, AIDS, Acquired immunodeficiency syndrome, Aged, Immunity, cellular, Antiretroviral therapy, highly active

## Abstract

**Objective:**

To characterize the epidemiological profile of patients aged 50 years or older diagnosed as HIV/AIDS, in a Specialized Service of the Public Health System.

**Methods:**

A retrospective cohort study using secondary data from medical records in the period 2014 to 2018. Sociodemographic and clinical characteristics, and features related to treatment adherence were organized in a database. Quantitative variables were expressed as mean (or median) ± standard deviation (or interquartile range), and categorical variables expressed as number and percentage of patients. The Kaplan-Meier method was applied to assess the probability of overall specific survival.

**Results:**

Of the 241 initially eligible patients, 149 patients were evaluated, registering 19 deaths in the studied period. There was a predominance of males aged 50-59 years, with severe immunodeficiency upon admission (29.7%), and with a CD4+ T lymphocyte count below 200 cells in 62 (46.3%) of patients. Elderly people aged 61 or over were more adherent. There was an increase in the CD4+ T lymphocyte count by an average of 139.63 in the first 6 months, and 50.51 from the first 6 months to 12 months of follow-up, with an average increase in the first 12 months of 157.63 cells. Specific overall survival in the period was 85%.

**Conclusion:**

Patients older than 50 years had an immune response and no viral load detection in the 12-month period, deserving further studies to improve survival.

## INTRODUCTION

Population aging is occurring rapidly all over the world and, following this trend, data from the *Instituto Brasileiro de Geografia e Estatística* (IBGE)^(1)^ suggest the elderly population in Brazil amounts to 28 million people or about 13% of total population. Aging is associated with increased adverse health risks, particularly in groups of people living with the same disease, such as infection by the human immunodeficiency virus (HIV).^(2)^

In the 1980´s, HIV infection was considered a disease of young individuals. However, the epidemiological data changed, the virus spread significantly among the elderly, with an increasing number of older people (50-year-old and over) living with the virus. This change was due to the longer survival achieved after the advent of highly active antiretroviral therapy, turning the focus to chronic disease management.^(3)^

The World Health Organization (WHO) and the Joint United Nations Programme on HIV/AIDS (UNAIDS) estimate that, of the 40 million people living with HIV/AIDS in the world, approximately 7.5 million are aged 50 and over and, in the context of HIV infection, these people are considered to be elderly. This is due to characteristics of this group, usually similar to those of individuals 5 to 10 years older, because the virus accelerates aging - including aging of the immune system.^(4)^ Chronologically, the WHO definition of elderly patients in developing countries is people aged 60 and over.

The prognosis for patients initiating treatment, in the modern era of antiretroviral therapy, is excellent, with a life expectancy close to that of same-age controls.^(5)^ Factors associated with poorer treatment evolution include a low CD4+ T cell count nadir, first diagnosis close to the age of 50 years and over, and transmission by intravenous drug use.^(5-7)^

Older patients are usually diagnosed at advanced stages of HIV infection, and several factors, including failure of health care professionals to suspect the disease in this population, as well infection symptoms being associated with many age-related conditions,^(8,9)^ have been implicated in this delayed diagnosis.

Prompt diagnosis and early onset of treatment are vital, considering the immune system of older adults tends to respond slower, compared with that of younger individuals. Also, good adherence to the therapy can postpone or prevent the onset of immunodeficiency.^(10,11)^ Likewise, maintenance of undetectable HIV viral load reduces the risk of sexual transmission of HIV to marginal levels, which makes preventive treatment a safe option for serodiscordant couples.

A current challenge has been adherence to antiretroviral system, defined as effective use of the prescribed drugs, since inappropriate or insufficient administration (skipped doses, wrong days or times) contributes to emergence of resistance to drug therapy, compromising clinical outcomes.^(12-14)^ Several promoters of non-adherence to drug therapy have been described, including factors related to regimen, patient, and health system. Some factors, such as polypharmacy, alcohol and drug abuse, and depression are frequently found in older patients, and will negatively affect adherence, in addition to individual differences, even though preliminary data have pointed to a more favorable behavior.^(12,14)^

In view of the above, specific measures focusing on prevention, diagnosis and treatment adherence by older patients should be incorporated into the management of chronic infection, which is a complex and dynamic phenomenon.

## OBJECTIVE

To identify the clinical-epidemiological profile of HIV/AIDS patients, aged 50 and over, seen at a Specialty Care Service of the Brazilian Public Health System. The prevalence of comorbidities, the level of cell immunodeficiency, adherence to treatment at month 6 and month 12, and their comparison with patients aged over and under 60 years, in addition to group mortality and survival indicators, were assessed.

## METHODS

A retrospective cohort study of adults diagnosed with HIV and AIDS at the Specialty Care Service (SAE) of the Health Surveillance Center (CVS) of the city of Juiz de Fora (MG). This outpatient facility services local patients and also those from 102 surrounding cities. The service is composed of a multidisciplinary team with infectious disease physicians, registered nurses and nurse technicians, dentists, psychologists and social workers, in addition to pharmacists from the Antiretroviral Drug Dispensing Unit (UDM - *Unidades Dispensadoras de Medicamentos*).

This study is a descriptive and exploratory analysis of secondary data from open and closed medical charts, and its inclusion criteria were patients aged ≥50 years, living with HIV and AIDS, who initiated medical care between January 2014 and December 2018.

Of the 19 deaths occurring during the study period, 13 were from AIDS-related causes, three from non-AIDS-related causes, and three from unidentified causes. The vital status of patients at the end of follow-up was verified in the medical chart and through the mortality information system (SIM - *Sistema de Informação sobre Mortalidade*) with data provided from the Municipal Epidemiological Surveillance Service.

Variables retrieved in patient charts included sociodemographic characteristics (sex, age, education level, race, marital status, drug use and transmission routes) and variables relative to health conditions and lifestyle (presence of manifestations of moderate or advanced immunodeficiency, as per the Clinical Protocols and Therapeutic Guidelines – PCDT, and comorbidities), in addition to clinical characteristics (CD4+ T cell count, twice-yearly viral load testing, number of visits and drug dispensations by the pharmacy).

Through the Laboratory Test Control System of the National Network for CD4+/CD8+ Cell Count and HIV Viral Load Testing (SISCEL - *Sistema de Controle de Exames Laboratoriais*) data were obtained on the CD4+ T cell counts using the FACScalibur™ Multitest flow cytometer,^(15)^ and HIV viral load values quantified by nucleic acid amplification using the Abbott RealTime HIV1 assay,^(16)^ which detects from 40 to 10 million copies. Cases of transient viremia or viral blips are defined as detection of low levels of viral RNA, typically between 50 and 200 copies.

Information on the antiretroviral drugs prescribed, as well as the number of pickups at month 6 and month 12, were retrieved from the Drug Logistics Control System (SICLOM - *Sistema de Controle Logístico de Medicamentos)*.

Eligible patients were categorized into three groups based on the classification of the Centers for Disease Control and Prevention (CDC), which emphasizes the importance of the CD4+ T cell count for follow-up of HIV-infected patients as follows: higher than or equal to 500cells/mm^3^, between 200-499cells/mm^3^ and lower than 200cells/mm^3^.^(15)^

Adherence to antiretroviral therapy was estimated through verification of the frequency of monthly dispensations by the pharmacy, considering as adherent patients who used 95% of prescribed doses,^(14)^ at months 6 and 12. For this purpose, an indicator was created establishing that up to five pickups at month 6 and ten pickups at month 12 would classify the patient as non-adherent.

Study data entry and analysis were organized in a database using Microsoft Excel and SPSS software, version 21. Quantitative variables were expressed as mean (or median) ± standard deviation (or interquartile range) and categorical variables as number and percentage of patients. The χ^2^ test was used for comparison of proportions.

The group’s proportional lethality and mortality indicators were calculated. To assess specific survival, the date of the first record at the Specialty Service was considered as the start of the survival period, and the deaths identified until the end of follow-up were treated as failures (information obtained by queries to the Mortality Information System of the city). Cases in which patients remained alive until the end of follow-up and those lost to follow-up (on December 31, 2020) were censored. The method proposed by Kaplan-Meier was used to assess the cohort’s survival probabilities.

This study was submitted to and approved by the Research Ethics Committee of the *Universidade Federal de Juiz de Fora* (UFJF), under CAAE: 11094919.3.0000.5147 and opinion # 3.379.925, and the need for an Informed Consent Form (ICF) was waived, according to resolution 466/2012 of the National Health Council, item IV.8.

## RESULTS

Of the 241 patients eligible for this study, 92 were excluded, of which 21 were from private practices, 66 were erroneously enrolled, four from other organizations and one with duplicate enrollment, for a total of 149 patients effectively enrolled, corresponding to 9.6% of the total newly diagnosed patients admitted between 2014 and 2018. There was a predominance of males (62.4%), age range between 50 and 59 years (65.8%), self-referring as white (57.7%), with 53.7% of patients with incomplete primary education or illiterate. In respect to the marital status, 82.5% of patients were not married or cohabiting. As for the use of drugs, the most frequent was tobacco (37.0%), followed by alcohol (34.2%) and Cannabis (8.2%).

The comorbidities reported in medical records of the study group included hypertension in 28.9% of patients, depression in 15.8% and diabetes in 10.3%. Variable grades of immunodeficiency were detected as early as on admission, with 29.7% of cases considered as severe immunodeficiency, and 23.6% as moderate immunodeficiency. CD4+ T cell counts inferior or equal to 200cells/mm^3^ were found in 46.3% of sample. This profile is described in table 1.

**Table 1:** Characteristics of users of the Specialty Care Service of the Brazilian Public Health System aged 50 years and over

Characteristics	n (%)
Age, years	
50-54	55 (36.9)
55-59	43 (28.9)
60-64	29 (19.5)
>65	22 (14.8)
Sex	
Female	56 (37.6)
Male	93 (62.4)
Race	
White	86 (57.7)
Pardo	32 (21.5)
Black	31 (20.8)
Education level	
Illiterate	8 (5.4)
Primary School	22 (14.8)
Secondary School	35 (23.5)
Further education	12 (8.1)
Incomplete Primary School	72 (48.3)
Form of transmission	
Sexual	85 (98.8)
Parenteral	1.0 (1.2)
Comorbidities	
Hypertension	43 (29.5)
Depression	23 (15.8)
Diabetes	15 (10.3)
Renal injury	5 (3.4)
Hepatitis C	5 (3.4)
COPD	4 (2.7)
Marital status	
Single	55 (36.9)
Separated or divorced	37 (24.8)
Widowed	31 (20.8)
Cohabiting	16 (10.7)
Married	10 (6.7)
Addiction	
Tobacco	27 (37.0)
Alcohol	25 (34.2)
Cannabis	6 (8.2)
Cocaine	5 (6.8)
Crack	4 (5.5)
Antiretroviral therapy	
TDF+3TC+EFZ	89 (61.8)
TDF+3TC+DTG	37 (25.7)
TDF+3TC+ATV/r	5 (3.5)
Other	13 (9.0)
Disease characteristics	
Asymptomatic immunodeficiency	69 (46.6)
Moderate immunodeficiency	35 (23.6)
Severe immunodeficiency	44 (29.7)
Baseline CD4+	
Under 200	62 (46.3)
201-499	40 (29.9)
>500	32 (23.9)
CD4+ at month 6	
Under 200	18 (21.4)
201-499	40 (47.6)
> 500	26 (31.0)
CD4+ at month 12	
Under 200	20 (23.3)
201-499	34 (39.5)
> 500	32 (37.2)

COPD: chronic obstructive pulmonary disease; TDF+3TC+EFZ: tenofovir + lamivudine + efavirenz; TDF+3TC+DTG: tenofovir + lamivudine + dolutegravir; TDF+3TC+ATV+r: tenofovir + lamivudine + atazanavir/ritonavir.

In respect to antiretroviral therapy and association of drugs, between 2014 and 2016, the tenofovir + lamivudine + efavirenz combination was the preferred regimen. After 2017, the combination of tenofovir + lamivudine and the new integrase inhibitor, dolutegravir, became the predominant regimen for patients initiating treatment; other alternative regimens are still used in cases of contraindication, adverse events or virologic failure.

As for monthly dispensation of antiretrovirals by the drug dispensing unit (UDM), 80 patients (56.7%) had six or more drug pickups in the first 6 months of treatment. At month 12, 81 (60%) users had more than 11 pickups and were considered adherent according to the adherence indicator defined for this study.

Table 2 shows the clinical results (adherence to drug treatment and monthly visits, in addition to viral load testing) per age group. Adherence was significantly higher among patients aged 60 and over at month 12. There was also an age-related difference in CD4+ T cells, with counts over 500cells/mm^3^ after 6 months of treatment in only 9.1% of patients aged 60 years and over.

**Table 2:** Association between age group and clinical characteristics of users of the Specialty Care Service of the Brazilian Public Health System aged 50 years and over

Variable	Total/lines	50-60 years 73.2% (109) n (%)	>61 years 26.8% (40) n (%)	p value
Adherence – month 6				
Yes	79	51 (50.0)	28 (73.7)	0.013
No	61	51 (50.0)	10 (26.3)	
Adherence – month 12				
Yes	80	54 (55.1)	26 (72.2)	0.078
No	54	44 (44.9)	10 (27.8)	
Viral load – month 6				
0-40	80	56 (75.7)	24 (77.4)	0.113
Blip	13	7 (9.5)	6 (19.4)	
>200	12	11 (14.9)	1 (3.2)	
Viral load – month 12				
0-40	92	67 (83.8)	25 (83.3)	0.912
Blip	9	6 (7.5)	3 (10.0)	
>200	9	7 (8.8)	2 (6.7)	
CD4+ – baseline value				
0-200	62	43 (44.3)	19 (51.4)	0.798
201-499	40	30 (30.9)	10 (27.0)	
>500	32	24 (24.7)	8 (21.6)	
CD4+ – month 6				
0-200	18	13 (21.0)	5 (22.7)	0.019
201-499	40	25 (40.3)	15 (68.2)	
>500	26	24 (38.7)	2 (9.1)	
CD4+ – month 12				
0-200	20	16 (25.8)	4 (16.7)	0.481
201-499	34	22 (35.5)	12 (50.0)	
>500	32	24 (38.7)	8 (33.3)	
Number of appointments – month 6				
0-2	63	49 (45.8)	14 (36.8)	0.350
>3	82	58 (54.2)	24 (63.2)	
Number of appointments – month 12				
0-4	69	51 (50.0)	18 (51.4)	0.884
>5	68	51 (50.0)	17 (48.6)	

Immune recovery, shown by an increment in the CD4+ T cell count over time resulted in an average gain of 139.63 at month 6, and 50.51 between month 6 and month 12, with an average increase of 157.63 cells at month 12.

**Figure 1:**
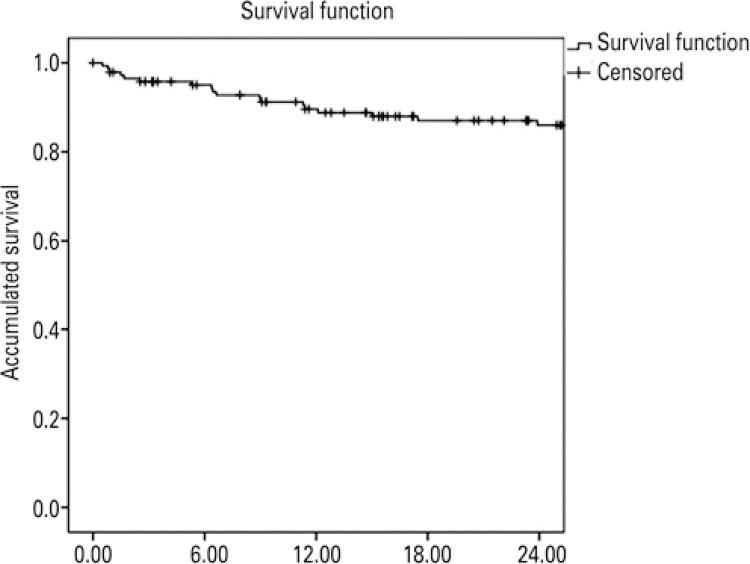
Specific survival of users of the Specialty Care Service of the Brazilian Public Health System

A total of 19 patients in the study group died between 2014 and 2018, with a mean age of 58 years, and with most of the deaths occurring in 2017. AIDS was confirmed as the primary cause of death in 13 cases. Therefore, AIDS lethality in the group was 8%, proportional mortality (considering confirmed causes) was 68%, and 24-month specific survival was 84.1%, as shown in figure 1.

## DISCUSSION

HIV infection is characterized by continuous loss of CD4+ T cells leading to immunodeficiency, opportunistic infections and death.^(17)^ A paradigm shift has changed the idea of the disease being fatal, and the results of antiretroviral therapy have led to the disease being treated as a chronic condition, with infected patients living longer and aging with a life expectancy similar tho that of the general population.^(5)^

There was a predominance of male patients aged between 50 and 59 years in this sample, mostly single or separated and with lower education levels, as well as with sexual transmission as the most common transmission route. The data point to a change in the age pyramid in recent decades, with an increasing number of older patients infected. Although the epidemic affects more young adults, older patients account for significant percentages of new cases.^(6)^ Sexuality must be considered an integrating element of life for individuals at different life stages. However, a low education level affects the understanding of information relative to infection and prevention, contributing to increased vulnerability of this population.^(8,9)^

People living with HIV can undergo accelerated aging, and those aged 50 years and over are considered elderly.^(5)^ An inflamed basal state, due to increased intestinal permeability and bacterial translocation caused by the virus, contributes to the prevalence of degenerative diseases typically related with aging^(18)^ and not related to AIDS in younger ages.^(2)^ In this study, 29.5% of patients had hypertension, and tobacco and alcohol use were the habits most often reported by the subjects in this sample, which are risk factors for atherosclerosis in HIV, in addition to the fact that older patients have an increased cardiovascular risk irrespective of antiretroviral therapy.^(19)^

The association of lower-toxicity antiretroviral drugs and drug-drug interactions can help in managing chronic conditions and geriatric syndromes and, in this regard, starting in 2017, an increase was seen in the use of the dolutegravir-containing regimen (previously, the predominant regimen was tenofovir + lamivudine + efavirenz), according to the Clinical Protocols and Therapeutic Guidelines (PCDT - *Protocolos Clínicos e Diretrizes Terapêuticas*) of the Ministry of Health. However, is worth highlighting that, in older patients, quality of life is more important than exposure to new regimens with potential side effects.^(14)^

The rate of severe and moderate immunodeficiency in this study was 29.7% and 23.6%, respectively, with 46.3% of patients on admission with CD4+ counts under 200 cells and high viral load. This status predisposes to faster disease progression, lower therapeutic success, and higher mortality after diagnosis, according to certain authors.^(20,21)^

The factors that determine CD4+ T cell response are partially elucidated; however, advanced age, long-standing disease and low baseline cell counts are risk factors for incomplete immune recovery.^(22)^ It is worth noting that, at month 6, there was an average increase of 139.63 cells. Between month 6 and month 12, there was an increase of 50.51 cells. At month 12, there was an average increase of 157.63 cells.

It is known, however, that the final clinical evolution also depends on factors related with good adherence and tolerance to antiretrovirals.^(23)^ Some studies have found that, in older patients, immune reconstitution is incomplete, in spite of treatment and undetectable viral loads.^(18,23)^ Viral load monitoring is the most important tool in the follow-up of patients who respond to antiretroviral therapy to assess.^(24)^ Older patients usually show higher treatment adherence.^(23,25)^

In this study, when it comes to adherence to treatment and monthly visits, it was significantly higher among patients aged 60 and over at month 12, which is beneficial, because it eliminates the risk of sexual transmission, since many do not adhere to preventive in spite of the fact they reach undetectable viral loads.^(12,20)^ On the other hand, a study comparing adherence among older and young patients did not show any difference between the groups.^(26)^

A higher education level and the use of simpler drug regimens promote greater adherence.^(21)^ However, use of drugs, particularly alcohol, management of comorbidities, depression and polypharmacy challenge the maintenance of adequate treatment in older patients.^(27)^

Of the deaths occurred during the study period, 13 were related with AIDS, with a proportional mortality rate of 68%. Since the advent of antiretroviral therapy, HIV-related mortality rates have been decreasing.^(28)^ Consequently, life expectancy is increasing worldwide,^(3,12)^ as well as age-related comorbidities.^(29)^

This study has some limitations inherent to retrospective studies, due to the use of secondary data and the limited information available in medical records, as well as patients lost to follow-up. On the other hand, the information collected showed that the elderly have specific needs, and caring for their health requires intervention by a multidisciplinary team to strengthen the bond and ensure sustained treatment. Since this is a reference service for Juiz de Fora (MG) and its surroundings, studies like this should further enhance the importance of team work and quality of care delivered.

## CONCLUSION

In the cohort of patients aged 50 years and over seen at the HIV/AIDS Specialty Care Service of a health care hub city in the Southeast of Brazil, higher adherence and viral load suppression were found in patients aged 61 years and over. After initiation of antiretroviral therapy, the sample showed an average gain of 157,63 CD4+ T cells in the first 12 months of treatment. The proportional mortality of the group was significant during the study period.

Identifying HIV/AIDS in the elderly is important. Diagnostic delay leads to patients with advanced disease and negative prognostic impact. Health care professionals must be attentive to prevention measures for sexually transmitted infections, and the dialogue about sexual activity in later life must be encouraged. Likewise, checking for potential risk factors of non-adherence to the therapy is vital for effective disease management in this population.
